# Reamed versus unreamed intramedullary nailing for the treatment of femoral fractures: A meta-analysis of prospective randomized controlled trials

**DOI:** 10.1097/MD.0000000000004248

**Published:** 2016-07-22

**Authors:** A-Bing Li, Wei-Jiang Zhang, Wei-Jun Guo, Xin-Hua Wang, Hai-Ming Jin, You-Ming Zhao

**Affiliations:** Department of Orthopaedics, The Second Affiliated Hospital of Wenzhou Medical University, Wenzhou, Zhejiang, China.

**Keywords:** femoral shaft fracture, meta-analysis, randomized controlled trials, reamed intramedullary nailing, unreamed intramedullary nailing

## Abstract

**Background and objective:**

Intramedullary nailing is commonly used for treating femoral shaft fractures, one of the most common long bone fractures in adults. The reamed intramedullary nail is considered the standard implant for femoral fractures. This meta-analysis was performed to verify the superiority of reamed intramedullary nailing over unreamed intramedullary nailing in fractures of the femoral shaft in adults. Subgroup analysis of implant failure and secondary procedure was also performed.

**Methods:**

Electronic literature databases were used to identify relevant publications and included MEDLINE (Ovid interface), EMBASE (Ovid interface), and the Cochrane Central Register of Controlled Trials (CENTRAL; Wiley Online Library). The versions available on January 30, 2016, were utilized. Only human studies, which were designed as randomized controlled clinical trials, were included. Two authors independently evaluated the quality of original research publications and extracted data from the studies that met the criteria.

**Results:**

Around 8 randomized controlled trials involving 1078 patients were included. Reamed intramedullary nailing was associated with shorter time to consolidation of the fracture (SMD = –0.62, 95% CI = –0.89 to –0.35, *P* < 0.00001), lower secondary procedure rate (OR = 0.25, 95% CI 0.10–0.62, *P* = 0.003), lower nonunion rate (OR = 0.14, 95% CI = 0.05–0.40, *P* < 0.01), and lower delayed-union rate (OR = 0.19, 95% CI = 0.07–0.49, *P* < 0.01) compared to unreamed intramedullary nailing. The 2 groups showed no significant differences in risk of implant failure (OR = 0.50, 95% CI 0.14–1.74, *P* = 0.27), mortality risk (OR = 0.94, 95% CI 0.19–4.68, *P* = 0.94), risk of acute respiratory distress syndrome (ARDS; OR = 1.55, 95% CI 0.36–6.57, *P* = 0.55), or blood loss (SMD = 0.57, 95% CI = –0.22 to 1.36, *P* = 0.15).

**Conclusion:**

Reamed intramedullary nailing is correlated with shorter time to union and lower rates of delayed-union, nonunion, and reoperation. Reamed intramedullary nailing did not increase blood loss or the rates of ARDS, implant failure, and mortality compared to unreamed intramedullary nailing. Therefore, the treatment of femoral fractures using reamed intramedullary nailing is recommended.

## 1 Introduction

Femoral shaft fractures comprise 5% to 6% of long bone fractures in adults, making them one of the most common fractures. Fractures of the femur are very frequently seen in patients who suffered multiple trauma or high-energy injury.^[1]^ The femur is an important weight-bearing bone and the improper treatment of a femoral fracture can result in deformity or dysfunction of the lower limb. Reamed intramedullary nailing (RIN) is considered the standard method of treatment for femoral fractures.^[2–5]^ The advantages of RIN include higher biomechanical stability,^[6]^ rapid fracture healing,^[7]^ and lower frequency of secondary procedure.^[7,8]^ However, several articles questioned whether or not the medullary cavity should be reamed. Opponents of reaming pointed out that reaming can decrease bone blood flow in the diaphysis^[9–11]^ and may cause bone necrosis^[12]^ and emboli.^[9,13]^ Reaming may also increase blood loss^[14–16]^ and operative time.^[14,15]^ This meta-analysis was performed to verify the superiority of reamed intramedullary nailing over unreamed intramedullary nailing in fractures of the femoral shaft in adults. In addition, we performed subgroup analysis of implant failure and secondary procedures. Although this systematic review was in progress, 2 similar meta-analyses were published.^[17,18]^ However, these meta-analyses did not include the articles published between 2011 and January 2016; thus, this review is more comprehensive.

## 2 Materials and methods

This study was performed with guidance from the Cochrane Handbook for Systematic Reviews of Interventions and the Preferred Reporting Items for Systematic Reviews and Meta-Analyses statement.^[19,20]^ As the present meta-analysis was performed based on previous published studies, ethical approval and patient consent were not required.

### 2.1 Inclusion and exclusion criteria

The criteria for inclusion were: (i) human studies that were designed as randomized controlled clinical trials, (ii) studies had to perform a comparison of RIN and URIN for the treatment of femoral shaft fractures, (iii) all participants were adults with mature skeletons, and (iv) when there was more than 1 study from the same center using the same protocol, the study with the longest follow-up was used. Exclusion criteria included: (i) pathological fractures, (ii) studies of fractures in animals or children, and (iii) nonrandomized studies, review articles, conference abstracts, biomechanical studies, or case reports.

### 2.2 Search strategy and study selection

We searched electronic literature databases for all relevant studies published prior to January 30, 2016. The databases used were MEDLINE (Ovid interface), EMBASE (Ovid interface), and the Cochrane Central Register of Controlled Trials (CENTRAL; Wiley Online Library). The search was performed without language restrictions, but was limited to human studies. The search strategies are shown in Table 1.

**Table 1:**
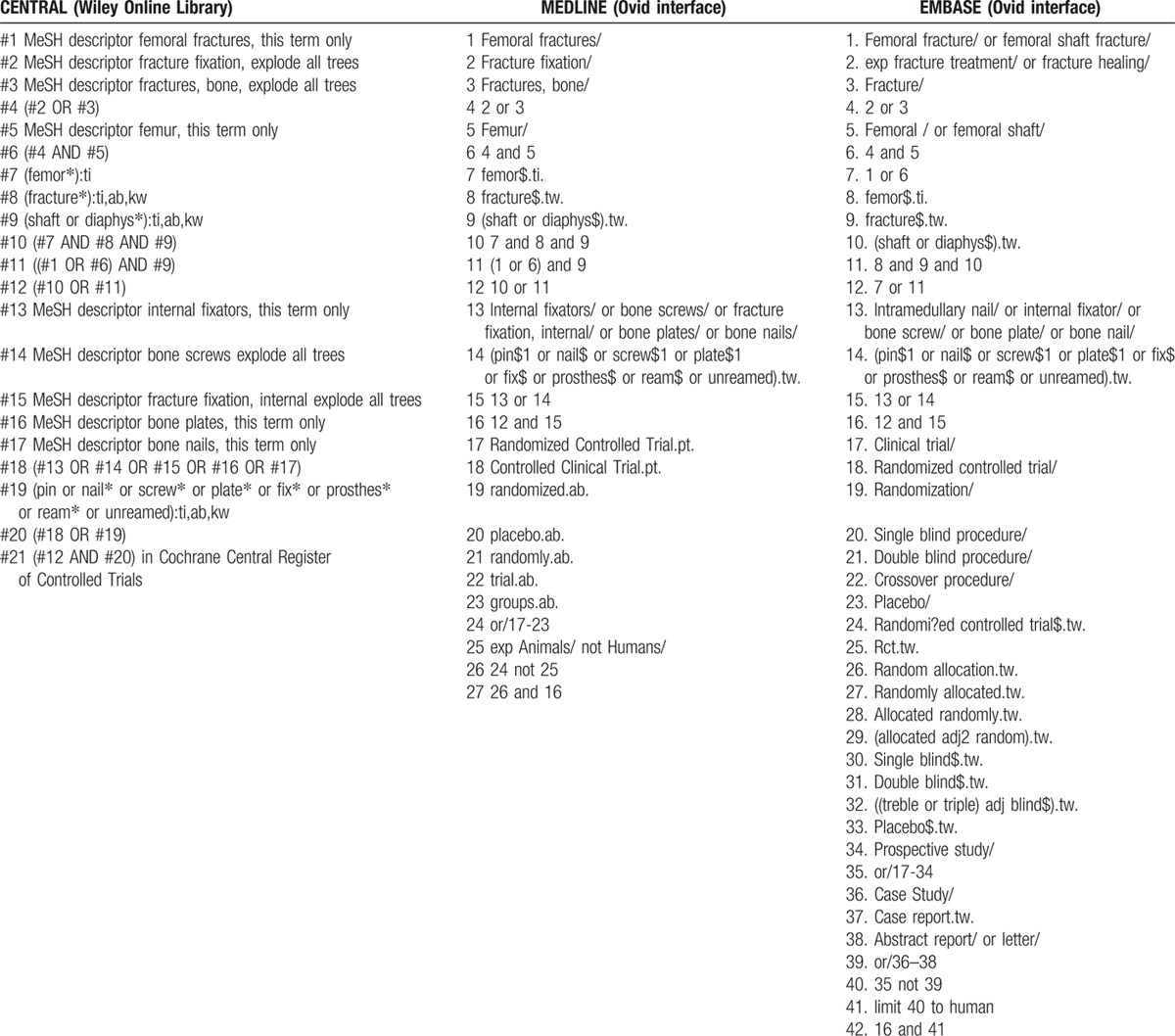
Search strategy.

The function of “related article” was also used to expand the search. In addition, we also manually searched references from review articles to supplement the electronic database search.

### 2.3 Data extraction

Two authors (ABL and HMJ) independently extracted the following data: the first author's last name, publication year, country, study follow-up duration, sample size, characteristics of patients, interventions, and post-operative complications. The third author (WJG) checked the agreement between the extracted information. If necessary, the primary authors were contacted to provide additional data.

### 2.4 Risk of bias assessment

All potential articles were independently assessed for methodological quality by 2 reviewers (ABL and WJZ) and any conflict was resolved by means of discussion with the third independent reviewer (YMZ). The reviewers assessed the risk of bias of included studies according to the Cochrane Handbook for Systematic Reviews of Interventions: random sequence generation; allocation concealment; double blinding of participants and personnel; blinding of outcome assessment; incomplete outcome data addressed; selective reporting; or other bias. The judgments of reviewers of bias were “low risk,” “high risk,” or “unclear risk.”

### 2.5 Statistical analysis

We used odd ratio (OR) as the effect measure of dichotomous outcomes, with 95% confidence intervals (CI). *I*^*2*^ statistic was used to evaluate the statistical heterogeneity. *I*^*2*^ > 50% was considered significantly statistical heterogeneity.^[21]^ A fixed effects model and 95% confidence intervals (CI) were used, and a random-effect model was considered if there was significant heterogeneity. Statistical analyses were conducted using the RevMan 5.3.5 software (The Nordic Cochrane Center, Denmark). *P* < 0.05 was considered statistically significant.

## 3 Results

### 3.1 Included studies

**Figure 1:**
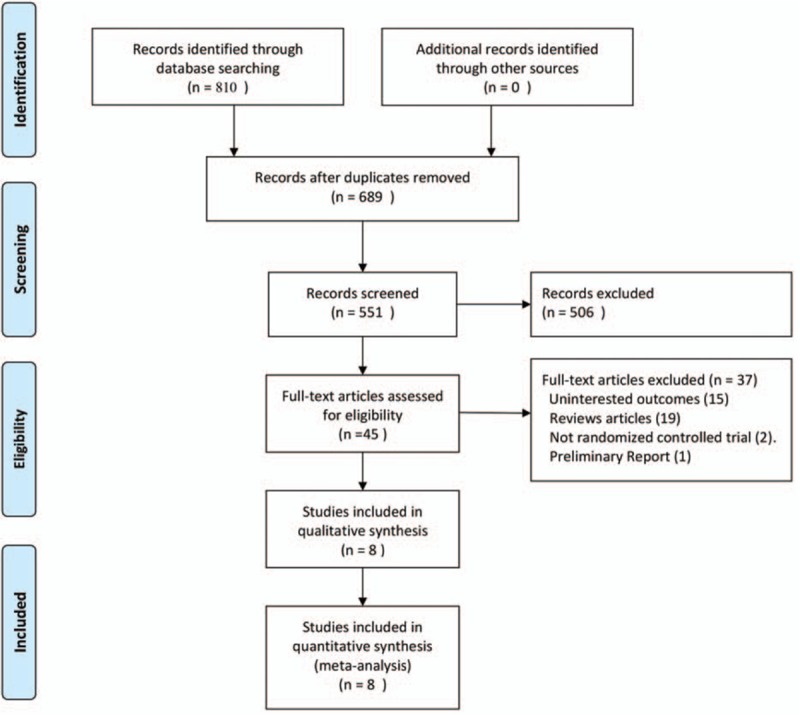
Flowchart of article selection for inclusion.

A total of 810 potentially relevant articles were identified from the databases, and 506 studies were excluded after screening of the title and abstract. A total of 45 full-text articles were assessed for eligibility. Of these, 2 were excluded as not randomized clinical trials; 15 trials were excluded due to the uninteresting outcomes; 19 studies were excluded as reviews articles; and 1 study was excluded because it was a preliminary report.^[22]^ The remaining 8 articles^[7,8,14–16,23–25]^ were included in this meta-analysis. Of these 8 studies, 2 were multicenter, randomized, controlled clinical trials and 6 were from a single investigational site and studied patients with femoral fractures. The selection of articles for inclusion is presented in Fig. 1.

The total number of participants studied was 1078 (541 in the RIN group and 537 in the URIN group) individuals; the studies were performed in various countries with no obvious discrepancies in baseline demographics between the RIN and URIN groups, and the individuals enrolled in all 8 studies were basically homogeneous. A summary of selected studies is shown in Table 2.

**Table 2:**
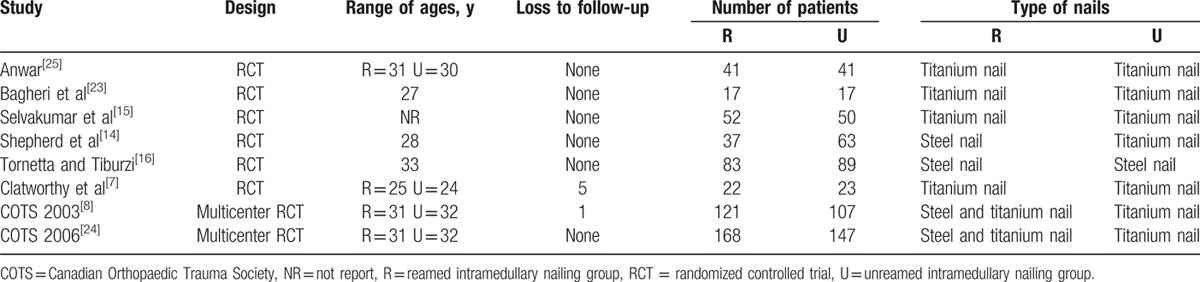
Characteristics of the included studies.

### 3.2 Risk of bias assessment

**Figure 2:**
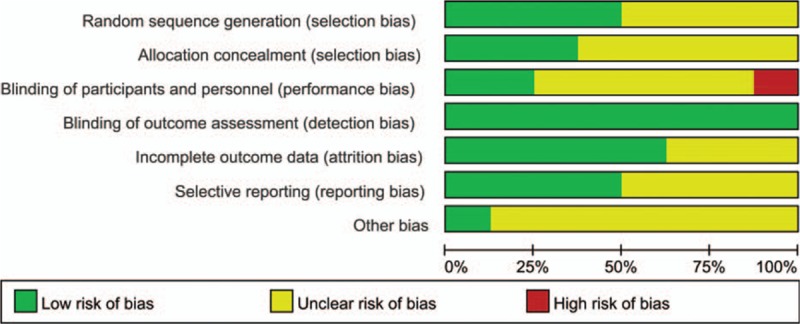
Risk of bias graph: review authors’ judgments about each risk of bias item presented as percentages across all included studies.

**Figure 3:**
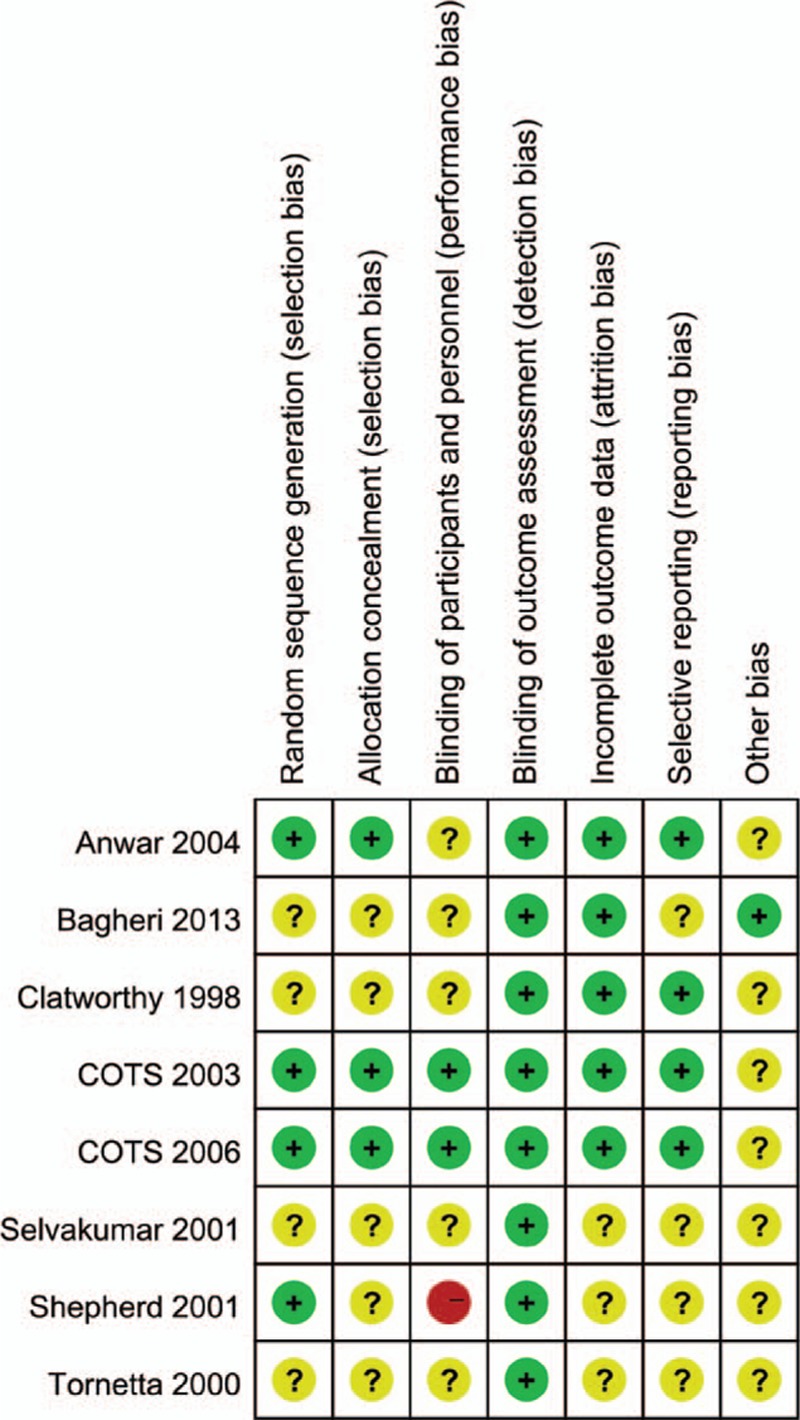
Risk of bias summary: review authors’ judgments about each risk of bias item for each included study.

The risk of bias of included studies is shown in Fig. 2 and summarized in Fig. 3. The randomization technique was mentioned in 4 trials,^[8,14,24,25]^ and information of allocation concealment was not provided for 5 studies.^[7,14–16,23–25]^ Because there was no difference in the postoperative radiological data between the 2 groups; thus, the term “blinding of outcome assessment” was assessed as “low risk” for all 8 studies.

### 3.3 Clinical outcomes

Due to different definitions of complications for the included studies, we did not perform a meta-analysis of overall complications. Thus, only the major adverse events including the incidence of nonunion, delayed-union, fixation failure, infection, acute respiratory distress syndrome (ARDS), and mortality were embedded into the meta-analysis for evaluation. Five studies^[7,14–16,23]^ reported the incidence of implant failure, with a low frequency in both groups.

**Figure 4:**
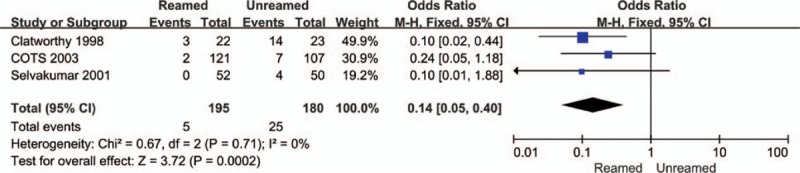
Forest plot showing nonunion rate of reamed intramedullary nailing and unreamed intramedullary nailing.

**Figure 5:**
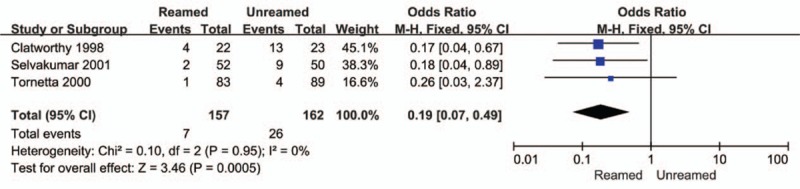
Forest plot showing delayed-union rate of reamed intramedullary nailing and unreamed intramedullary nailing.

**Figure 6:**
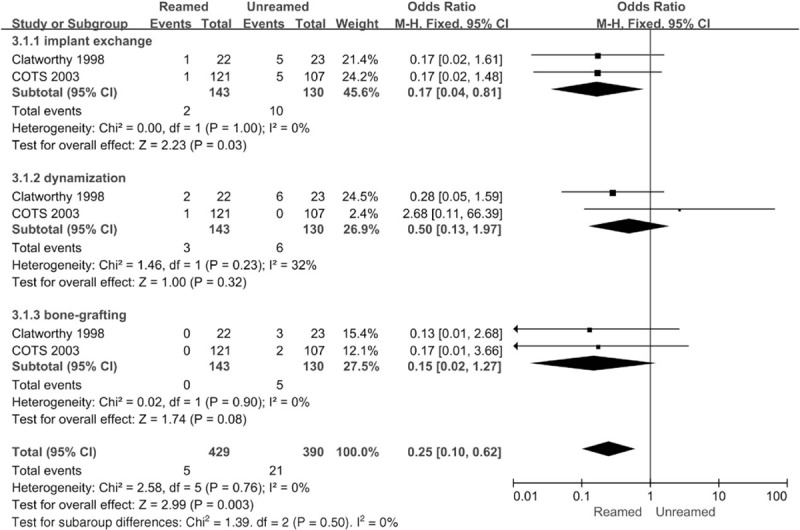
Forest plot showing subgroups analysis of secondary procedure of reamed intramedullary nailing and unreamed intramedullary nailing.

**Figure 7:**
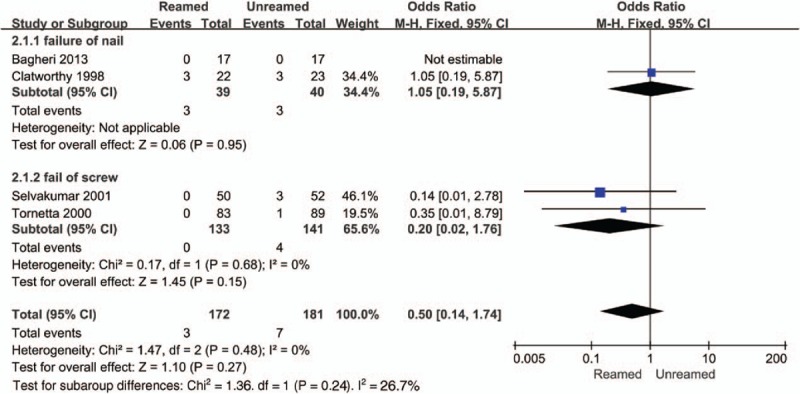
Forest plot showing subgroups analysis of implant failure of reamed intramedullary nailing and unreamed intramedullary nailing.

**Figure 8:**
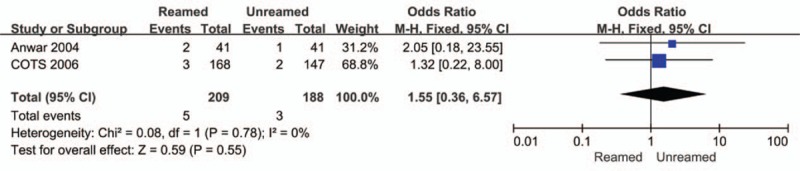
Forest plot showing the ARDS rate of reamed intramedullary nailing and unreamed intramedullary nailing.

**Figure 9:**
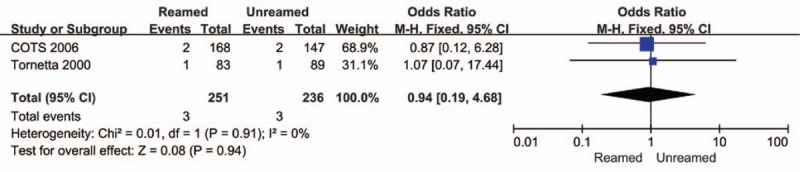
Forest plot showing mortality rate of reamed intramedullary nailing and unreamed intramedullary nailing.

The incidences of nonunion (OR = 0.14, 95% CI = 0.05–0.40, *P* < 0.01, Fig. 4) and delayed-union (OR = 0.19, 95% CI = 0.07–0.49, *P* < 0.01, Fig. 5) were significantly higher in the URIN group. Reamed intramedullary nailing showed a significantly lower rate of secondary procedures when compared to URIN (OR = 0.25, 95% CI 0.10–0.62, *P* = 0.003 Fig. 6). The subgroup analysis demonstrated a higher risk of implant exchange (OR = 0.17, 95% CI = 0.04–0.81, *P* = 0.03, Fig. 6) of patients treated with URIN. However, there were no significant differences between the groups for the risk of bone grafting (OR = 0.15, 95% CI = 0.02–1.27, *P* = 0.08, Fig. 6) or dynamization (OR = 0.50, 95% CI = 0.13–0.97, *P* = 0.32, Fig. 6). Our subgroup analysis found no differences in nail failure (OR = 1.05, 95% CI = 0.19–5.87, *P* = 0.95, Fig. 7) or screw failure (OR = 0.20, 95% CI = 0.02–1.76, *P* = 0.015, Fig. 7). Additionally, the 2 groups showed no significant differences for risk of ARDS (OR = 1.55, 95% CI = 0.36–6.57, *P* = 0.55, Fig. 8) or mortality (OR = 0.94, 95% CI = 0.19–4.68, *P* = 0.94, Fig. 9).

**Figure 10:**
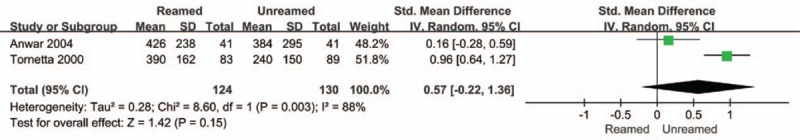
Forest plot showing blood loss of reamed intramedullary nailing and unreamed intramedullary nailing.

**Figure 11:**
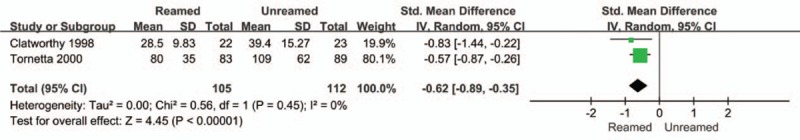
Forest plot showing time to union of reamed intramedullary nailing and unreamed intramedullary nailing.

The differences in blood loss between the 2 groups were not significant (SMD = 0.57, 95% CI = –0.22 to 1.36, *P* = 0.15 Fig. 10). There was obvious statistical heterogeneity in these results (Chi^2^ = 8.6, *I*^2^ = 88%, *P* = 0.003 Fig. 10). There was also apparent decreased time to union for the RIN group (SMD = –0.62, 95% CI = −0.89 to −0.35, *P* < 0.00001 Fig. 11).

## 4 Discussion

The purpose of this study was the evaluation of the relative merits of RIN versus URIN in the treatment of femoral shaft fractures. This analysis indicated that RIN improved the union rate of fractures, decreased the time to union, and decreased the incidence of non-union or delayed-union; furthermore, it did not correlate with increased blood loss or the risks of ARDS, implant failure, and mortality. Although there was no obvious difference in implant failure between the 2 groups, the incidence of secondary procedures (implant exchange) was higher in the URIN group than in the RIN group.

Intramedullary nailing is the standard treatment for fractures of the femoral shaft in adults. Titanium alloy or stainless steel is often used as the material for nails. The biomechanical properties of the materials can affect fracture healing. Titanium alloy has a lower elastic modulus, which is close to the human bone elasticity and is more biocompatible than stainless steel. Therefore, the insertion of a Titanium nail enhances callus formation and shortens time to bone union, resulting in a high healing rate. In addition, compared with unreamed intramedullary nailing, reamed intramedullary nails of larger diameters can be inserted and their fatigue strength or bending stiffness is higher. We noted a shorter time to union for the RIN group and the time to union was shorter in patients in whom a Titanium nail was inserted. However, Trompeter and Newman^[26]^ reported no significant difference in time required for union and failure rates for the 2 materials. Due to the relatively small size of samples in this study, these results should still be interpreted with caution. The differences between titanium or steel nails should be further verified by additional prospective randomized clinical trials with larger sample sizes.

El Maraghy et al^[11]^ reported that reaming might destroy the nutrient artery and decrease bone blood flow in the diaphysis. Based on this, researchers predicted that bone blood supply that was reduced due to reaming damage could influence fracture healing and increase the risk of infection.^[27]^ However, this is not supported our analysis. Instead, we found that fracture union was significantly slower in the URIN group and the incidences of nonunion and delayed-union were significantly lower in the RIN group. The blood supply of long bones was well-characterized by Rhinelander who showed that the medullary arteries supply the inner two-thirds of the cortex, and that the outer third is supplied by the periosteal vasculature via its soft tissue attachments to the bone.^[28]^ When a fracture occurs, the medullary vessels are disrupted, leading to 50% to 70% necrosis of the cortex near the fracture site. Some researchers have speculated that the debris produced by reaming may include osteoblasts^[29]^ and multipotent stem cells^[30]^ placed at the fracture site to act as an autologous bone graft.^[31]^ Reaming may damage the blood supply of the inner cortical bone, but in response, the periosteal blood flow can increase 6-fold, which may stimulate fracture healing.^[32]^

The treatment methods for delayed-union or nonunion after bone fracture include dynamization, bone grafting, implant exchange, and electrostimulation.^[33]^ Clatworthy et al^[7]^ concluded that fracture stability was an important determinant of rapid union. A larger nail is inserted into the medullary cavity after reaming to improve cortical contact and provide greater stability.^[34,35]^ Grundnes et al^[36]^ reported a tightly fitting nail increased the periosteal reaction. However, it is still unclear if RIN, thought to provide increased mechanical stability, will reduce the need for implant exchange compared to the URIN group. By subgroup analyses, our study found no obvious differences in the risks of bone grafting and dynamization between the 2 groups, but the risk of implant exchange was lower for the RIN group. Our findings indicate RIN may provide greater stability and reduce the risk of implant exchange. In the treatment of femoral fractures, economic costs must also be considered. Secondary procedures are very expensive and are correlated with high rates of complications and mortality. Our study found a high rate of secondary procedures in the URIN groups, which would require higher costs. Therefore, the treatment of femoral fractures using reamed intramedullary nailing is recommended.

From a technical point of view, implant failures include clinical screw or nail failure. Screw failures are more common than nail failures.^[37]^ Screw failure and nail failure correlated with a higher risk of implant failures. However, we observed no obvious differences between RIN and URIN groups for the risk of implant failures. By subgroup analyses, we also found that reamed intramedullary nailing, in contrast with the URIN group, did not increase the incidences of screw failure and nail failure. Consistent with the conclusions of a separate analysis, our results suggest that the time to union, the presence of an open wound, and the configuration of the fracture were the most critical predisposing factors of implant failures. This conclusion should be further verified by additional prospective randomized clinical trials with larger sample sizes.^[7]^

Various clinical studies have suggested that reaming increased intramedullary pressure of the femur, releasing more bone marrow components and fat emboli into pulmonary circulation compared to treatment without reaming.^[38]^ Potential clinical adverse events include FES, ARDS, multiple organ dysfunction syndrome (MODS), and sudden death. However, the rates of FES and ARDS were low in our analysis. Only 8 cases of ARDS were reported in these studies. No cases of FES were documented in any of the included studies.^[7,8,14–16,23–25]^ We found no obvious differences in ARDS or mortality rate between the 2 groups.

Alho et al^[39]^ concluded that the risk of infective complications was higher in the RIN group when compared to the URIN group. However, some studies found that there was no obvious discrepancy in infection rates between the groups.^[40,41]^ In all included studies, the rate of infective complications was low and only 2 studies reported infective complications.^[8,23]^ In 1 study, there were 4 cases with infection (3 superficial and 1 deep infection).^[8,23]^ However, the number of infections for each group was not reported. Another study described a superficial infection in the RIN group.^[8,23]^ Due to insufficient data, we did not perform meta-analysis of the rate of infection.

Unreamed femoral nailing may have a potential advantage of less blood loss. Less blood loss can reduce the need for transfusion, eliminating complications of blood transfusion and reducing costs.^[42,43]^ The reduced intraoperative blood loss will benefit elderly patients with multimorbidity, as other diseases may take precedence. However, we did not observe significant differences in blood loss for the 2 groups. Due to high heterogeneity, these results should be interpreted with caution. In practice, surgeons usually estimate the blood loss and different assessment methods of intraoperative bleeding were used in different hospitals, such as collection from a plastic bag taped to the surgical drapes, suction drain, or from the weight of swabs. These differences may mask any differences in blood loss for these methods. That could explain the statistic significant difference of heterogeneity.

Most studies compared reamed and unreamed intramedullary nails for closed femoral fractures, but a few studies compared the reamed and unreamed intramedullary nails for treatment of both closed femoral fractures and open femoral fractures. However, any complications that occurred were not distinguished by fracture type, precluding our ability to perform subgroup analysis according to different types of fractures.

Several limitations of this analysis should be noted. First, this article only focused on the rates of nonunion, delayed-union, mortality, implant failure, reoperation, ARDS, blood loss, and the time to union. We did not assess outcomes such as functional results or satisfactory outcomes or time to definitive treatment because these parameters were not always reported or were reported in various forms and not directly comparable. Second, this study did not evaluate quantitative outcome measures such as weight-bearing time, operative time, and hospital stay. Finally, only 8 studies with 1078 participants were included in the review, which might weaken the reliability of this meta-analysis. Despite these limitations, our quantitative evaluation of the rates of complications, blood loss, and the time to union provide an important foundation for surgical treatment decisions.

## 5 Conclusion

Reamed intramedullary nailing is correlated with shorter time to union and lower rates of delayed-union, nonunion, and reoperation. Reamed intramedullary nailing did not increase blood loss or the rates of ARDS, implant failure, and mortality compared to unreamed intramedullary nailing. Therefore, the treatment of femoral fractures using reamed intramedullary nailing is recommended.

## Acknowledgments

The authors thank all the anonymous reviewers and editors for their helpful suggestions on the quality improvement of our paper.
